# History of Pregnancy Loss as a Risk Factor for Myocardial Infarction

**DOI:** 10.7759/cureus.17288

**Published:** 2021-08-18

**Authors:** Atif Hussain Sarwar, Balvender Singh, Sindhu Kishore, FNU Priyanka, Ahmed Ali, FNU Pariya, Parkash Bachani, Sidra Naz, Simra Shahid, Faizan Shaukat

**Affiliations:** 1 Internal Medicine, Ghulam Muhammad Mahar Medical College, Sukkur, PAK; 2 Internal Medicine, Jinnah Sindh Medical University, Karachi, PAK; 3 Internal Medicine, Shaheed Mohtarma Benazir Bhutto Medical University, Larkana, PAK; 4 Infectious Disease, University of Louisville, Louisville, USA; 5 Internal Medicine, Liaquat University of Medical and Health Sciences, Jamshoro, PAK; 6 Internal Medicine, University of Health Sciences, Lahore, PAK; 7 Internal Medicine, Dow University of Health Sciences, Karachi, PAK

**Keywords:** pregnancy, cardio vascular disease, abortion, myocardial infarction, high-risk pregnancy, miscarriage

## Abstract

Introduction

There are few cardiovascular risk factors that are unique to females, such as after menopause, lipid profiles change unfavorably. Another risk factor that might be associated with an increased risk of cardiovascular diseases in women is the incidence of miscarriages and abortions. In this study, we will determine the association between the previous history of pregnancy loss and myocardial infarction (MI).

Methods

This case-control study was conducted from December 2019 to January 2021. We enrolled 600 female patients with a confirmed diagnosis of MI from the outpatient department (OPD) of the cardiology and internal medicine unit of a tertiary care hospital in Pakistan. Another 600 female participants without the diagnosis of MI were enrolled from the OPD as the control group. Participants were asked about the history of pregnancy, including the number of miscarriages, abortions, and stillbirths.

Results

Participants with myocardial infarction had experienced greater than one miscarriage compared to participants without MI (25.1% vs. 13.6%; p-value: <0.0001). Similarly, participants with MI had significantly more participants with stillbirth compared to participants without MI (12.0% vs. 6.66%; p-value: 0.0017).

Conclusion

Pregnancy loss is associated with MI in the future. Women with a history of pregnancy loss must undergo regular cardiovascular screening to protect themselves from cardiovascular events.

## Introduction

Cardiovascular disease (CVD) affects both males and females and is seen as one of the prime causes of morbidity and mortality globally. Its prevalence is relatively higher in the South Asian population compared to the rest of the world [1]. Recent data suggest a surge in the prevalence and mortality ratio of coronary heart disease, specifically among the female gender, below 55 years of age [2]. 

There are various common risk factors for men and women, such as cigarette smoking, hypertension, diabetes mellitus (DM), and obesity [3]. However, there are a few risk factors that are unique to women. After menopause, lipid profiles change unfavorably, with an increase in the levels of low-density lipoprotein cholesterol and a decrease in the levels of high-density lipoprotein cholesterol [4]. Another risk factor that might be associated with an increased risk of CVD in women is the incidence of miscarriages and abortions. Horn et al. stated a significant association of pregnancy losses with comorbidities, such as hypertension, DM type 2, and deranged lipid profile; all of which can contribute to an increased risk of CVD [5].

Various studies have identified spontaneous abortion and stillbirth as risk factors for ischemic heart disease and stroke [6,7]. However, the pathogenesis leading to an increased risk of CVD remains considerably understudied. Globally, there is very limited data available on pregnancy loss as a risk factor for CVDs. Therefore, in this study, we will establish the association between pregnancy loss and myocardial infarction (MI).

## Materials and methods

This case-control study was conducted from December 2019 to January 2021. We enrolled 600 female patients with a confirmed diagnosis of MI from the outpatient department (OPD) of the cardiology and internal medicine unit of a tertiary care hospital in Pakistan. The diagnosis of MI was confirmed based on electrocardiogram findings, cardiac biomarkers, and clinical symptoms. ECG findings included changes in ST-segment, the appearance of Q waves or disappearance of S waves, cardiac biomarkers included raised troponin I and clinical symptoms included chest pain, shortness of breath, sweating, and nausea. Another 600 female participants without the diagnosis of MI were enrolled from the OPD as the control group. Both case and control group participants were enrolled via consecutive convenient non-probability sampling. Participant’s informed consent was taken before enrollment. The ethical approval was taken from the institutional review board before the enrollment of participants. Participants were informed that they have the right to withdraw their consent at any time during the study.

Age, history of smoking, existing comorbidities, such as DM and hypertension and family history for MI, was noted in a self-structured questionnaire. Participants were asked about the history of pregnancy, including the number of miscarriages, abortions, and stillbirths. 

Analysis of the data was done using the Statistical Package for Social Sciences® software, version 23.0 (SPSS; IBM Corp., Armonk, NY, USA). Continuous variables, such as age, were presented as mean and standard deviation (SD). Percentages and frequencies were calculated for categorical variables. Using a 95% confidence interval, the odds ratio was calculated via an online calculator MedCalc (MedCalc Software bv, Ostend, Belgium). A p-value of ≤0.05 was considered statistically significant.

## Results

The participants of both the MI and non-MI groups had a mean age of 53 ± 13. We found no significant difference in demographics between the participants of both groups (Table 1).

**Table 1:** Demographics of the participants BMI: body mass index, kg/m^2^: kilogram per meter square, MI: myocardial infarction, NS: nonsignificant, SD: standard deviation.

Characteristics	Participants with MI (n = 600)	Participants without MI (n = 600)	p-value
Age in year (mean ± SD)	53 ± 13	53 ± 13	NS
Hypertension	522 (87.0%)	512 (85.3%)	NS
Smoking	78 (13.0%)	71 (11.8%)	NS
Diabetes	222 (37.0%)	232 (38.6%)	NS
BMI greater than 25 kg/m^2 ^	201 (33.5%)	199 (33.1%)	NS
Family history of acute MI	52 (8.6%)	49 (8.16%)	NS

Participants with MI significantly reported more than one miscarriage compared to participants without MI (25.1% vs. 13.6%; p-value <0.0001). Similarly, participants with MI significantly reported more stillbirth compared to participants without MI (12.0% vs. 6.66%; p-value: 0.0017) (Table 2).

**Table 2:** Association of miscarriage, stillbirth, and medical abortion with MI MI: myocardial infarction.

Characteristics	Participants with MI (n = 600)	Participants without MI (n = 600)	Odds ratio	p-value
Participants with more than one miscarriage	151 (25.1%)	82 (13.6%)	2.12 (1.57-2.85)	<0.0001
Participants with stillbirth	72 (12.0%)	40 (6.66%)	1.90 (1.27-2.86)	0.0017
Participants with medical abortion	20 (3.33%)	16 (2.66%)	1.18 (0.61-2.20)	0.61

## Discussion

The MI group had significantly more participants with more than one miscarriage and stillbirth compared to the group of participants without MI. The results of our study are consistent with other studies, which proved similar results. Compared to the women who underwent abortions, the women with a history of spontaneous early miscarriages have been found to be at an increased risk of CVD [8]. Consistent with the results of our study, a two-fold increase in the risk of MI has been observed in Finnish pregnant women with a history of miscarriage [9].

It is known that estrogen in women has a protective effect on the cardiovascular system (CVS). However, reproductive history, which affects the levels of endogenous estrogen during a woman’s life, is not known to have an impact on CVD risk. The number of times a woman is pregnant and its duration plays an important role in determining how long a woman is exposed to a higher estrogen level. Therefore, contrary to the full-term pregnancy, an unsuccessful pregnancy exposes a woman to a lower level of estrogen; hence, not having enough protective effect on CVS [10]. On the other hand, one of the risk factors for pregnancy loss and pre-eclampsia are high homocysteine levels in early pregnancy [11]. The long-term adverse consequences of gestational hypertension and pre-eclampsia on cardiovascular events are widely known [12,13]. Therefore, a high homocysteine level is known to have an increased impact on CVD associated with miscarriage. Antiphospholipid antibodies (APLA) also play an important role in the CVD events associated with early pregnancy loss. Proteins present in the blood react with these antibodies that are bound to phospholipids in cell membranes. This results in serious complications, such as stroke, heart attack, and miscarriage. Tests for lupus anticoagulant and anticardiolipin antibodies, which are associated with the APLA syndrome, are highly recommended in women having consecutive early pregnancy losses [14,15].

The scientific rationale of this study is to highlight pregnancy loss as a risk factor for MI. Early pregnancy loss is associated with some serious complications, including stroke, CVD, etc. Therefore, to avoid such complications, regular monitoring and screening is important if a history of consecutive early pregnancy loss is present. To the best of our knowledge, this is the first study in the regional setting to study the association between pregnancy loss and cardiovascular events. However, there were few limitations in this study as well. First, since the study was conducted in a single institute, the sample size and diversity were limited. Secondly, since there was no official record of the number of miscarriages and pregnancy loss for most women, patients had to recall from their memory, so we might have recall bias in our study.

## Conclusions

Our study showed a positive association between MI and past incidences of pregnancy loss. Pregnancy loss is responsible for detrimental effects leading to atherosclerotic CVD. It is imperative that the pathogenesis and risk factor profile responsible for deteriorating outcomes in young women is studied thoroughly. It will help in better recognition of the prevalence of potential CVD risk factors in association with pregnancy loss and decrease the rate of morbidity and mortality in these women. Women with a history of pregnancy loss must undergo regular cardiovascular screening to protect themselves from cardiovascular events.
